# The Fungal Defensin Family Enlarged

**DOI:** 10.3390/ph7080866

**Published:** 2014-08-18

**Authors:** Jiajia Wu, Bin Gao, Shunyi Zhu

**Affiliations:** Group of Animal Innate Immunity, State Key Laboratory of Integrated Management of Pest Insects and Rodents, Institute of Zoology, Chinese Academy of Sciences, 1 Beichen West Road, Chaoyang District, Beijing 100101, China

**Keywords:** peptide antibiotic, gene duplication, exon-intron structure, cysteine-stabilized α-helical and β-sheet motif

## Abstract

Fungi are an emerging source of peptide antibiotics. With the availability of a large number of model fungal genome sequences, we can expect that more and more fungal defensin-like peptides (fDLPs) will be discovered by sequence similarity search. Here, we report a total of 69 new fDLPs encoded by 63 genes, in which a group of fDLPs derived from dermatophytes are defined as a new family (fDEF8) according to sequence and phylogenetic analyses. In the oleaginous fungus *Mortierella alpine*, fDLPs have undergone extensive gene expansion. Our work further enlarges the fungal defensin family and will help characterize new peptide antibiotics with therapeutic potential.

## 1. Introduction

Fungal defensin-like peptides (fDLPs) are emerging as attractive anti-infective agents due to their therapeutic efficacy, low toxicity and high serum stability [1,2]. On the basis of a combined analyses of sequence, structural, and phylogenetic data, we has identified seven fDLP families [2,3], in which three members (plectasin, micasin and eurocin), classified as ancient invertebrate-type defensins (AITDs) [1,2,4,5], have been structurally and functionally characterized. These fDLPs exhibit activity against several antibiotic-resistant clinical isolates with significant therapeutic potential [1,2,5,6]. Some efforts have been taken to improve antimicrobial efficacy and to reduce undesirable side effects of fDLPs. For example, an improved mutant of plectasin (NZ2114) is superior to two conventional antibiotics (vancomycin and daptomycin) in inhibiting methicillin-resistant *Staphylococcus aureus* (MRSA) with even more enhanced serum stability and extended *in vivo* half-life [7,8,9]. In this work, we describe 69 new fDLPs in terms of their sequences, structural characteristics, and phylogenetic relationship. This provides an array of candidates for development of new anti-infective agents against antibiotic-resistant human pathogens.

## 2. Discovery of New fDLPs

**Figure 1:**
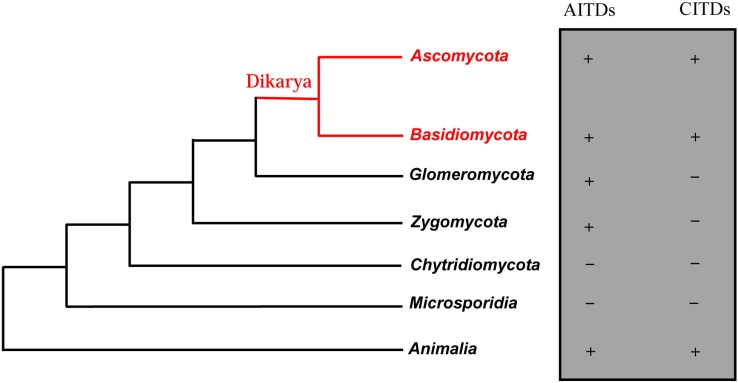
Phylogenetic distribution of fDLPs. The left: A parsimony tree of fungal species, animalia is used as an outgroup. This tree is a modification of the SSU and LSU r-RNA analyses of Lutzoni *et al*. for the fungal kingdom [10]. The right: “+” means presence and “−” means absence.

**Figure 2:**
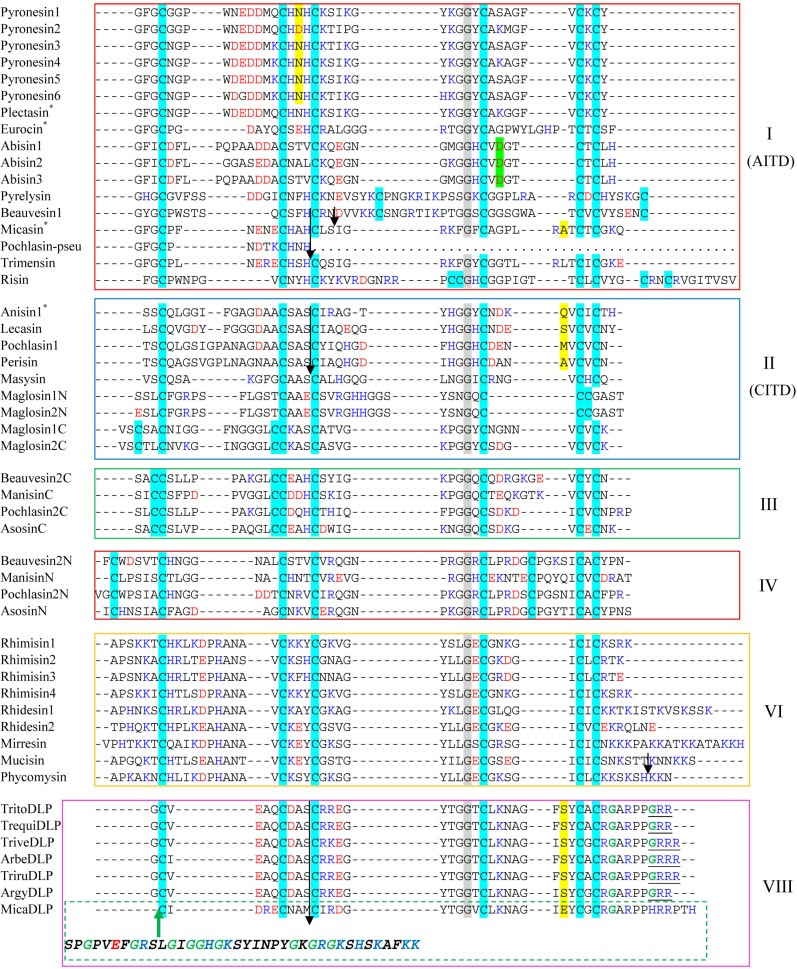
Multiple sequence alignment of fDLPs. Cysteines are shadowed in cyan. Conserved glycines are highlighted in grey. Negatively (D and E) and positively (R, K and H) charged residues are boldfaced in red and blue, respectively. Introns are shown by arrows (phase 0) or small boxes (green: phase 1, yellow: phase 2). Functionally characterized fDLPs were indicated by “*”. The N-terminal extension sequence in micaDLP belonging to the family fDEF8 is italicized. Defensins from *Pyronema omphalodes* have been predicted and investigated by RNA-seq [13]. Extra residues for C-terminal amidation are underlined once.

**Figure 3:**
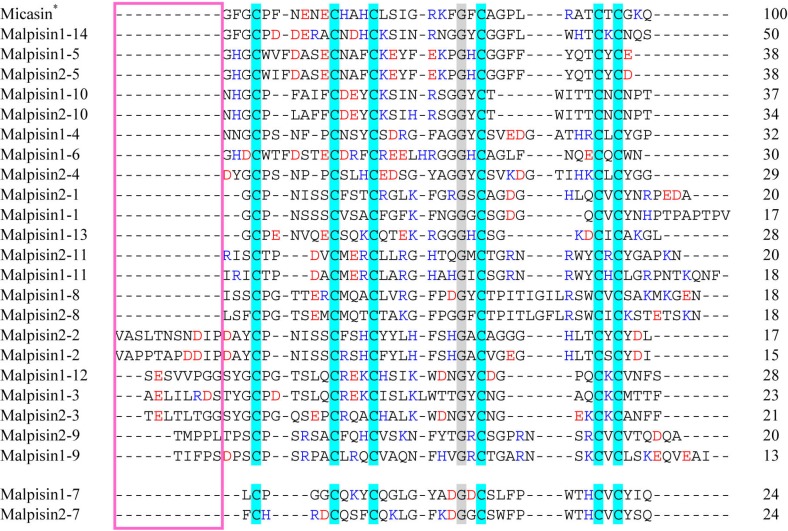
Multiple sequence alignment of malpisins. Color codes and symbol notes used here are the same as those in Figure 2. Pink box indicates the N-terminus of DLPs with variable length. Sequence identity (%) to micasin is shown on the right.

**Figure 4:**
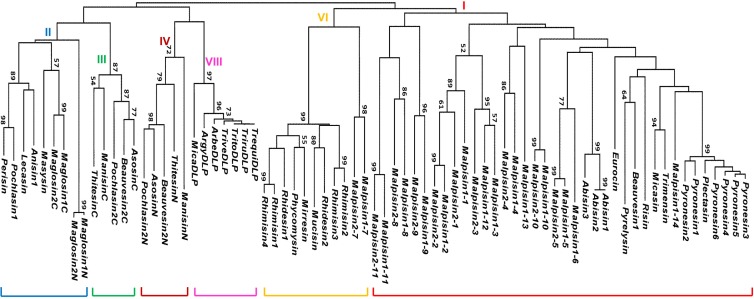
Phylogenetic tree of fDLPs. The tree was constructed from the aligned amino acid sequences presented in Figure 2 and Figure 3 with the neighbor-joining method. The numbers on nodes represent bootstrap values, and only values ≥50% are shown.

The database search strategy used here has been described previously [3]. Through an exhaustive search of 26 fungal species, we retrieved a total of 69 new fDLPs. As previously stated, overall this class of molecules exhibits a taxa-specific distribution pattern in the fungus kingdom, of which 21 fDLPs are derived from *Ascomycota*, 39 from *Zygomycota*, eight from *Basidiomycota* and one from *Glomeromycota*. In the basal fungi (*Microsporidia* and *Chytridiomycota*), no typical fDLP has been identified (Figure 1). The general features of these peptides are listed in Table 1 and Table 2. They can be grouped into six families based on sequence similarity, five of which are classified into the previously known families (fDEF1, fDEF2, fDEF3, fDEF4, and fDEF6) [3] (Figure 2 and Figure 3). This grouping is consistent with the phylogenetic analysis supported by high bootstrap values (Figure 4).

**Figure 5:**
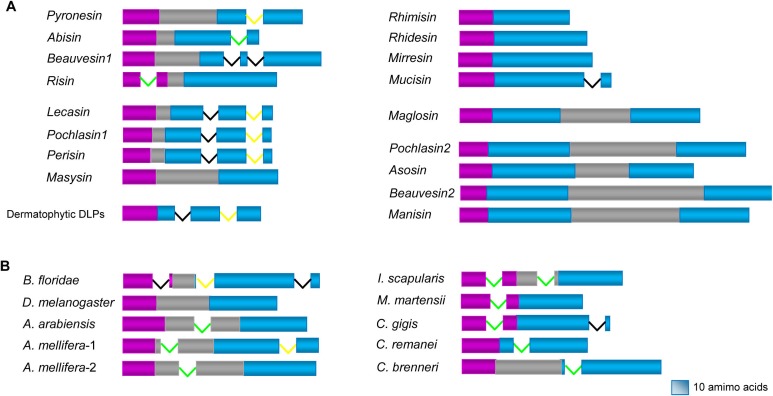
Comparison of precursor organization and exon-intron structures between fDLPs and animal defensins. (**A**) fDLPs; (**B**) Animal defensins. Signal, pro- and mature peptides are shown in pink, grey and blue, respectively. Intron phases are shown in the same colors as Figure 2. Representative animal defensins are derived from *Branchiostoma floridae*, *Drosophila melanogaster*, *Anopheles arabiensis*, *Apis mellifera*, *Ixodes scapularis*, *M. martensii*, *Crassostrea gigas*, *Caenorhabditis remanei*, and *C. brenneri*.

All the fDLPs characterized here have a signal peptide located in the N-terminus. In comparison with fDEF1 and fDEF2 that possess a propeptide located between signal and mature peptides, fDEF6 and fDEF8 lack a propeptide. Five precursors (maglosin, beauvesin2, manisin, pochlasin2 and asosin) could release two defensins from a single precursor after the removal of a spacer propeptide (Figure 5). The malpisin family from *Mortierell*a *alpine* exhibits two types of precursor organization: (1) the first type contains 10 members, all having a propeptides identified by its acidic feature and single or two basic amino acids at their ends as putative cleavage site of proprotein convertase [11]; (2) the second type contains 14 members that lack a propeptide and thus no further processing step is needed (Figure S1).

**Table 1:** Sources and characteristics of newly discovered non-*Mortierell*a fDLPs.

Name|Accession No.	Class	Species (phylum: subphylum: class)	Size	MW	NC
Pyronesin1|CATG01000243 (G)	fDEF1	*Pyronema omphalodes* (Ascomycota: Pezizomycotina: Pezizomycetes)	40	4317	+1.2
Pyronesin2|CATG01000243 (G)		*P. omphalodes*	40	4402	+0.2
Pyronesin3|CATG01000243 (G)		*P. omphalodes*	40	4389	+1.2
Pyronesin4|CATG01000243 (G)		*P. omphalodes*	40	4416	+2.2
Pyronesin5|CATG01000243 (G)		*P. omphalodes*	40	4375	+1.2
Pyronesin6|CATG01000243 (G)		*P. omphalodes*	40	4291	+2.4
Abisin1|AEOK01000166 (G)		*Agaricus bisporus* (Basidiomycota: Agaricomycotina: Agaricomycetes)	40	4097	‒3.8
Abisin2|AEOK01000166 (G)		*A. bisporus*	40	4097	‒3.8
Abisin3|AEOK01000166		*A. bisporus*	39	3926	‒2.8
Beauvesin1|ADAH01000714 (G)		*Beauveria bassiana* (Ascomycota: Pezizomycotina: Sordariomycetes)	52	5475	+2.9
Pyrelysin|GAJI01023341 (T)		*Pyrenochaeta lycopersici* (Ascomycota: Pezizomycotina: Dothideomycetes)	55	5858	+5.4
Risin|JAQX01005622		*Rhizophagus irregularis* (Glomeromycota: Glomeromycetes)	55	5972	+6.1
Trimensin|FG132536 (E)		*Trichophyton mentagrophytes* (Ascomycota: Pezizomycotina: Eurotiomycetes)	38	4156	+2.2
Lecasin|AWYC01000479	fDEF2	*Lecanosticta acicola* (Ascomycota: Pezizomycotina: Dothideomycetes)	42	4314	‒4.8
Pochlasin1|AOSW01002431		*Pochonia chlamydosporia* (Ascomycota: Pezizomycotina: Sordariomycetes)	43	4339	‒3.5
Perisin|AFRD01000258		*Periglandula ipomoeae* (Ascomycota: Pezizomycotina: Sordariomycetes)	43	4080	‒1.5
Masysin|CANK01000016		*Malassezia sympodialis* (Basidiomycota: Ustilaginomycotina: Exobasidiomycetes)	35	3432	+2.2
Maglosin1N|AAYY01000039 (G)		*Malassezia globosa* (Basidiomycota: Ustilaginomycotina: Exobasidiomycetes)	40	3980	+1.2
Maglosin2N|AAYY01000024 (G)		*M. globosa*	40	4022	+0.2
Maglosin1C|AAYY01000039 (G)		*M. globosa*	41	3910	+2.7
Maglosin2C|AAYY01000024 (G)		*M. globosa*	40	3835	+2.7
Beauvesin2C|ADAH01000123 (G)	fDEF3	*B. bassiana*	41	4243	+0.9
ManisinC|ADNJ01000735		*Metarhizium anisopliae* (Ascomycota: Pezizomycotina: Sordariomycetes)	41	4211	‒0.1
Pochlasin2C|AOSW01005877		*P. chlamydosporia*	41	4381	+0.2
AsosinC|BACA01000303		*Aspergillus sojae* (Ascomycota: Pezizomycotina: Eurotiomycetes)	38	4002	‒1.0
Beauvesin2N|ADAH01000123 (G)	fDEF4	*B. bassiana*	48	5067	+2.9
ManisinN|ADNJ01000735		*M. anisopliae*	46	4921	+0.2
Pochlasin2N|AOSW01005877		*P. chlamydosporia*	49	5185	+2.9
AsosinN|BACA01000303		*A. sojae*	49	5140	‒1.1
Rhimisin1|ANKS01000620	fDEF6	*Rhizopus microsporus* (Zygomycota: Mucoromycotina: Mucorales)	45	4867	+10.0
Rhimisin2|ANKS01000620		*R. microsporus*	44	4638	+3.4
Rhimisin3|ANKS01001486		*R. microsporus*	44	4768	+1.5
Rhimisin4|ANKS01001486		*R. microsporus*	45	4811	+8.0
Rhidesin1|AACW02000043		*Rhizopus delemar* (Zygomycota: Mucoromycotina: Mucorales)	55	5885	+10.4
Rhidesin2|AACW02000259		*R. delemar*	48	5270	+0.5
Mirresin|AZYI01000143		*Mucor irregularis* (Zygomycota: Mucoromycotina: Mucorales)	60	6424	+13.4
Mucisin|AOCY01001156 (G)		*Mucor circinelloides* (Zygomycota: Mucoromycotina: Mucorales)	53	5548	+2.2
Phycomysin|EX863311 (E)		*Phycomyces blakesleeanus* (Zygomycota: Mucoromycotina: Mucorales)	50	5342	+9.4
TritoDLP|ACPI01000196 (G)	fDEF8	*Trichophyton tonsurans* (Ascomycota: Pezizomycotina: Eurotiomycetes)	41	4323	+3.7
TrequiDLP|ABWI01000729 (G)		*Trichophyton equinum* (Ascomycota: Pezizomycotina: Eurotiomycetes)	41	4323	+3.7
TriveDLP|ACYE01000402		*Trichophyton verrucosum* (Ascomycota: Pezizomycotina: Eurotiomycetes)	42	4403	+4.7
ArgyDLP|ABQE01000293		*Arthroderma gypseum* (Ascomycota: Pezizomycotina: Eurotiomycetes)	41	4247	+3.7
ArbeDLP|ABSU01000004		*Arthroderma benhamiae* (Ascomycota: Pezizomycotina: Eurotiomycetes)	42	4493	+4.7
TriruDLP|ACPH01000567 (G)		*Trichophyton rubrum* (Ascomycota: Pezizomycotina: Eurotiomycetes)	42	4479	+4.7
MicaDLP|ABVF01000093		*Arthroderma otae* (Ascomycota: Pezizomycotina: Eurotiomycetes)	43	4745	+3.2

*Note:* MW: molecular weight; NC (net charge) is estimated at pH 7.0 with protein calculation V3.4. “E” means peptides from the Expressed Sequence Tags (EST) database and “T” means peptides from the Transcriptome Shotgun Assembly (TSA) database. “G” means proteins currently annotated in the GenBank database as hypothetical proteins (http://www.ncbi.nlm.nih.gov/) [12].

**Table 2:** Sources and characteristics of the malpisin family.

Name|Accession No.	Organism	Scaffold (Contig)	Range	Size	MW	NC
Malpisin1-1|AZCI01001104	*Mortierella alpina* B6842	jtg7180000084593f_7180000084594f	55070–55405	41	4048	‒0.0
Malpisin1-2|AZCI01001104			55870–56127	48	5166	‒3.3
Malpisin1-3|AZCI01001104			56393–56635	45	5047	+0.7
Malpisin1-4|AZCI01001104			63869–64117	39	4117	‒1.0
Malpisin1-5|AZCI01000882		Contig 7180000084767	22045–22248	37	4259	‒2.8
Malpisin1-6|AZCI01000882			25851–26051	39	4543	‒3.5
Malpisin1-7|AZCI01000882			42456–42677	33	3624	‒1.0
Malpisin1-8|AZCI01000882			43573–43800	47	5078	+1.7
Malpisin1-9|AZCI01000882			45037–45261	48	5203	+3.0
Malpisin1-10|AZCI01000882			45559–45738	35	3914	‒0.0
Malpisin1-11|AZCI01000882			46707–46913	43	4941	+5.4
Malpisin1-12|AZCI01001135		jtg7180000084204f_7180000084205f_7180000084206f	135437–135676	44	4722	‒0.0
Malpisin1-13|AZCI01001084		jtg7180000084699f_7180000084700f	362415–362627	47	4919	‒1.8
Malpisin1-14|AZCI01001006		jtg7180000084769f_7180000084770f_7180000084771f_7180000084772f	179488–179673	38	4188	+0.2
Malpisin2-1|ADAG01001070	*Mortierella alpina* ATCC 32222	Contig 1070	9785–10114	39	4105	+1.0
Malpisin2-2|ADAG01001070			10532–10792	48	5187	‒2.3
Malpisin2-3|ADAG01001070			11052–11297	44	4783	‒0.0
Malpisin2-4|ADAG01001070			11773–12021	39	4052	‒1.8
Malpisin2-5|ADAG01000791		Contig 791	4894–5097	37	4259	‒2.8
Malpisin2-7|ADAG01000903		Contig 903	13145–13357	33	3899	+1.2
Malpisin2-8|ADAG01000903			14223–14450	47	5065	+1.7
Malpisin2-9|ADAG01000903			15634–15852	45	4917	+4.0
Malpisin2-10|ADAG01000903			16158–16337	35	3937	+0.2
Malpisin2-11|ADAG01000903			17264–17446	39	4531	+5.0

Peptides in fDEF8, all derived from dermatophytes, are characterized as a new family with a short N-terminus and an extra C-terminal extension rich in arginines, prolines and glycines (Figure 2). The C-terminal extension has been considered as a common mechanism for the complexity increase of some invertebrate antimicrobial peptides (AMPs). For example, the hymenopteran defensin-1 subfamily has an extended C-terminus relative to its ancestral defensin-2 subfamily by a so-called intron exonization-mediated mechanism [14,15]. It thus appears that fungal and invertebrate defensins both convergently evolved their C-termini. The extension of a C-terminal sequence via convergent evolution was also recently observed in interleukin 6 (IL-6), a class-I helical cytokine, of two leporids (*Oryctolagus* and *Pentalagus*) [16]. The presence of C-terminal Gly-Arg or Gly-Arg-Arg in some dermatophyte-derived fDLPs suggest that they may be amidated, as previously observed in some animal toxins, e.g., the *Mesobuthus* α-toxins [17]. Interestingly, the mature peptide of micaDLP is larger in size than that of other members in this family, as identified by an N-terminal extension of 38 amino acids (Figure 2). High content of glycines together with a cationic characteristic hints a putative antimicrobial role of this extended unit.

*M. alpine* is a saprophytic species of *Mucoromycotina*, known as an oleaginous fungus [18]. The draft genome sequences of two *M. alpina* isolates (B6842 and ATCC 32222) [18,19] provide a possibility to undertake comparative study of their fDLPs. We found that the *M. alpine* B6842 genome encodes 14 fDLPs (Figure 3) but only 10 were found in *M. alpine* ATCC 32222. The failure to detect the four homologs (*i.e.*, malpisin1-6, 1-12, 1-13, 1-14) in *M. alpine* ATCC 32222 could be due to the incompletely-assembled genome sequences. Our phylogenetic analysis divides all malpisins into fDEF1 and fDEF6 (Figure 4). Some malpisin members of fDEF1 extended their N-termini with diverse sequences and variable lengths (Figure 3).

## 3. Gene Duplication of fDLPs

**Figure 6:**
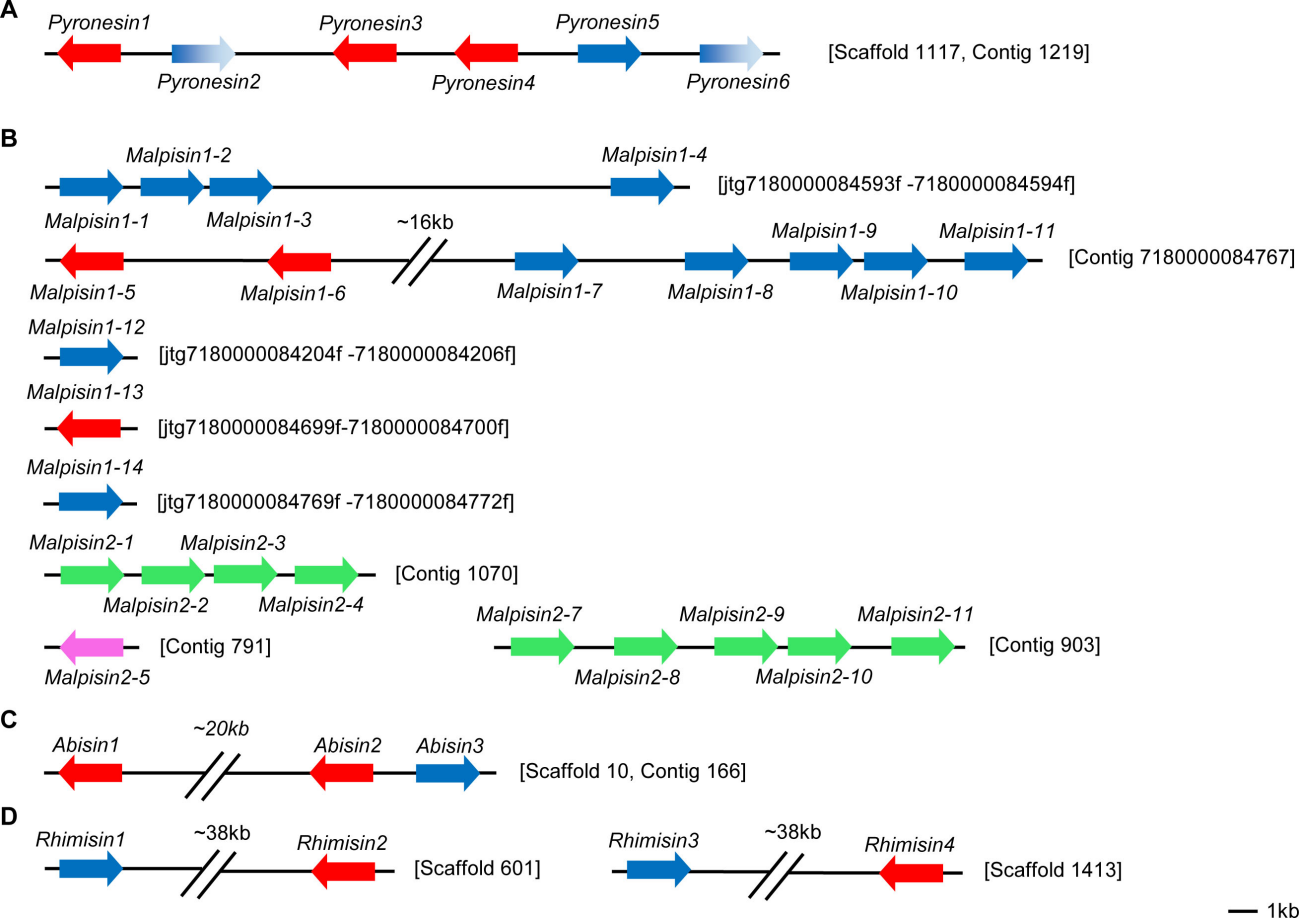
The arrangement of defensin genes in chromosomes. Color arrows refer to different orientation of the genes. **A** to **D** represent the genome location of defensins in four species: *Pyronema omphalodes*, *Mortierella alpine*, *Agaricus bisporus* and *Rhizopus microsporus*. Malpisins in *M. alpine* B6842 is indicated in red and blue while in pink and green in *M. alpine* ATCC 32222. Pseudogenes of pyronesins are shown in gradient blue.

Gene duplication extensively occurs in antimicrobial peptides from insects to humans [15,20,21]. In fungi, initial annotation of defense molecules of *Pyronema omphalodes* also identified gene duplication as a minor multigene family of fDLPs (herein termed pyronesin1 to pyronesin6) [13]. These fDLPs are highly similar to plectasin (Figure 6A). Our studies revealed new gene duplication event in other fungal species. Malpisin is a representative example of gene duplication. As mentioned previously, there are 14 and 10 members in *M. alpine* B6842 and *M. alpine* ATCC 32222, respectively. Malpisin1-1, 1-2, 1-3 and 1-4 are tandem located on one contig (jtg7180000084593f _7180000084594f), and malpisin1-5 to malpisin1-11 on another contig (Contig 7180000084767). In addition, malpisin1-12, malpisin 1-13 and malpisin 1-14 reside on other three contigs, as shown in Figure 6B. In *M. alpine* ATCC 32222, malpisin2-1 to malpisin2-4 are located on contig 1070 and malpisin2-7 to malpisin2-11 on contig 903. Only malpisin2-5 is located on contig 791.

In the widely cultivated mushroom *Agaricus bisporus*, there are three paralogous fDLPs (abisin1 to abisin3) (Figure 6C), two of which (abisin1 and abisin3) share completely identical amino acid sequences in the mature peptide region but exhibit four synonymous substitutions at the nucleotide level. In the *Pochonia chlamydosporia* paralogues, pochlasin1 is highly similar to CITDs and pochlasin2 possesses two defensin-domains. In addition, a putative pseudogene (herein named pochlasin-pseu) was also identified in scaffold 1191 and assigned to AITDs in view of its high sequence similarity to micasin in the first exon. Pochlasin1 and pochlasin-pseu share a conserved phase 0 intron within the α-helical region. The loss of the last two exons (2 and 3) results in the lack the last four cysteines involved in the Csαβ folding of a mature peptide (Figure 2).

Gene duplication also occurs in the *Mucorales-*derived fDLPs, which leads to four and two gene copies in *Rhizopus microsporus* (Figure 6D) and *R. delemar*, respectively. In a Neighbor-Joining (NJ) tree, rhimisin1 and rhimisin4 (*R. microsporus*) constitutes a single clade clustering with the other three fDLPs (rhidesin1 from *R. delemar*, phycomycin from *Phycomyces blakesleeanus* and mirresin from *Mucor irregularis*) whereas rhimisin2 and rhimisin3 (*R. microsporus*) cluster with rhidesin2 (*R. delemar*) and mucisin (*M. circinelloides*) (Figure 4), suggesting that the gene duplication event could have occurred in the ancestor of the *Mucorales* prior to their speciation.

## 4. Variable Gene Structures of fDLPs

Analysis of the exon-intron structures of the newly-discovered fDLPs revealed their variability that can be described as follows: (1) all the fDLPs retain the integrity of the signal peptide except risin (*Rhizophagus irregularis*) and malpisin1-1 (or malpisin2-1) that have a phase 1 or phase 0 intron disrupting their signal peptides; (2) all of the genes in fDEF8 and three genes in fDEF2 (*i.e.*, lecasin, pochlasin1 and perisin) have the same gene organization as previously identified dermatophytic defensins (micasin, arbesin, trivesin, tritosin and trirusin) and they contain two introns: the first intron (phase 0) disrupting the α-helical region; the second intron (phase 2) disrupting the c-loop; (3) the pyronesin and abisin multi-gene family in fDEF1 have only one intron disrupting either the α-helical or the c-loop region; (4) In addition to these intron-containing fDLP genes, there are some members without introns (Figure 2 and Figure 5).

The highly variable gene structures in fDLPs are reminiscent of invertebrate defensins that also exhibit diverse gene structures [22,23] (Figure 5 and Figure S2). Compared with invertebrate defensins of 5'-biased intron positions, introns of fDLPs occur preferentially in the 3'-end of the precursor-coded sequences. Because all eukaryotic Csαβ-type defensins are hypothesized to be originated from a common bacterial ancestor [24], it is reasonable to infer that considerable intron gains might have occurred in defensins from some eukaryotic lineages, and later they differentially lost in some specific species. Such a dynamic intron evolution thus shapes the biased intron location pattern between fDLPs and animal DLPs after the animal-fungi split. It is also worth mentioning that some recognizable orthologues of defensins in *Branchiostoma floridae* [25,26], the basal chordate amphioxus, also contain a phase 0 intron located in their c-loop (Figure 5 and Figure S2). Given a remote evolutionary distance between fungi and amphioxus, their intron position conservation could be a consequence of convergent insertion in a similar position due to the existence of “protosplice sites” [27,28]. However, the evolution via ancestral origin can be not completely ruled out in the case of the lack of gene structure information in many animal defensins from different lineages.

## 5. Conclusions

It is estimated that there are as many as 1.5 million species of fungi in this world. However, only a small fraction has been described and even fewer have been sequenced. To date, only about six hundred genomes were being sequenced or completely sequenced. Fungal genome project (FGP) allows us to systematically exploit peptide antibiotics instead of accidental discovery or complicated biochemical screening. This work sheds light on the persistent discovery of fDLPs from model fungal genome data. Despite this, in the lack of experimental data, it cannot be stated that all these fDLPs possess antibacterial function because in fact a classical insect-type fungal defensing - pechrysin was found to lack antibacterial activity [29] likely due to the absence of cationic residues on its molecular surface. In addition, anisin1, a DLP from *Aspergillus giganteus*, was found to be involved in the fitness of the species by linking stress signaling with developmental regulation [30]. Recent studies have also shown that although some peptides of fungal origin contain a similar defensin structure, they exhibit diverse or alternative biological functions beyond antimicrobial activity. An interesting overview is given by Hegedüs and Marx [31]. Therefore, further biochemical characterization of these newly-discovered fDLPs will help evaluate their potential as human medicines.

## Supplementary Files

Supplementary File 1
