# Supplementary material for: The Fungal Defensin Family Enlarged
Source: Pharmaceuticals (Basel). 2014 Aug 18;7(8):866–80. doi: 10.3390/ph7080866 (PMC4165938; doi:10.3390/ph7080866)

Supplementary Materials

Figure S1. Multiple sequence alignment of the malpisin family. Signal, pro- and mature peptides are boxed in pink, gray and blue. Color codes and symbol notes used here are the same as those in Figure 2. The arrow labels the position of a phase 0 intron.

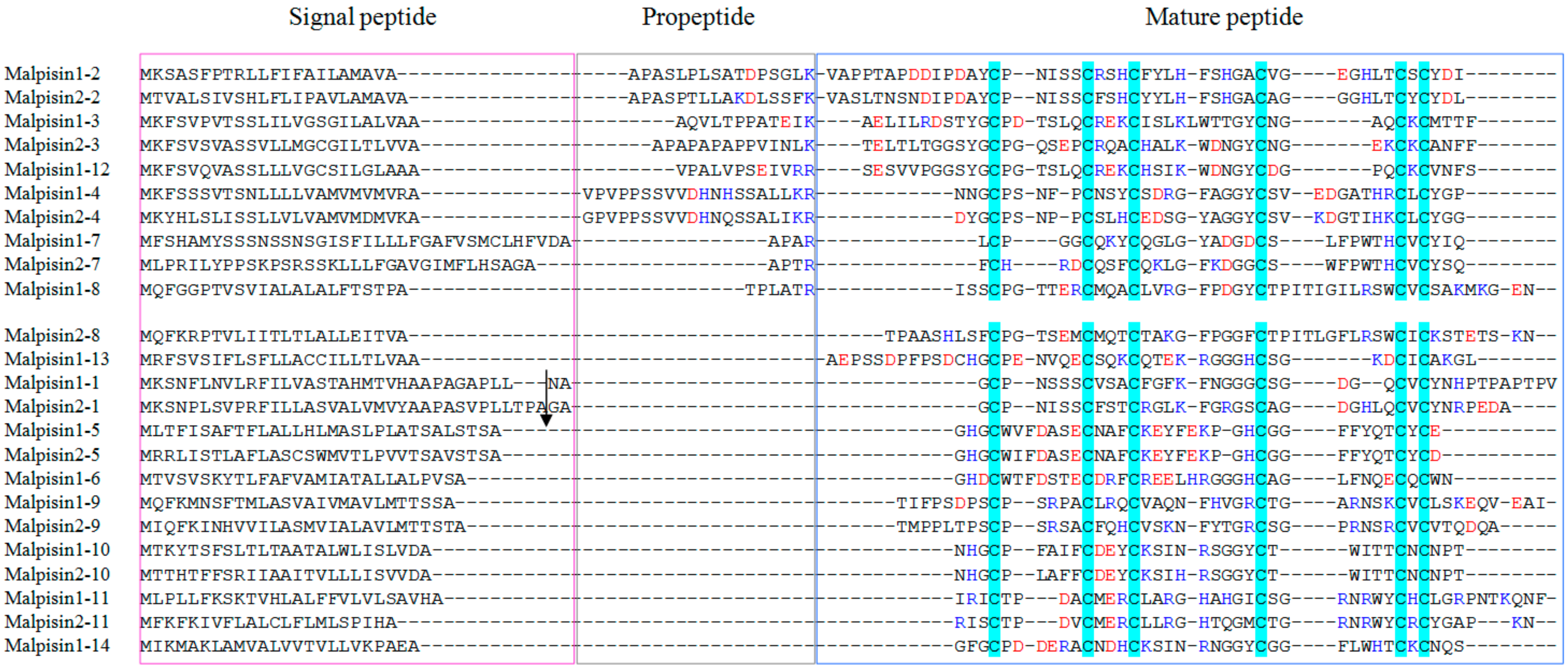

Figure S2. Multiple sequence alignment of animal defensins. Signal, pro- and mature peptides are boxed in pink, gray and blue, respectively. Color codes and symbol notes used here are the same as those in Figure 2. The GenBank accession numbers of the defensin sequences are *B. floridae* (XP_002596529), *D. melanogaster* (NP_523672), *A. arabiensis* (ABB00945), *A. mellifera*-1 (AADG06005896), *A. mellifera*-2 (AADG06003648), *I. scapularis* (DS766801), *M. martensii* (AYEL01078893), *C. gigas* (AFTI01007260), *C. brenneri* (ABEG02001428), *C. remanei* (AEM44806).

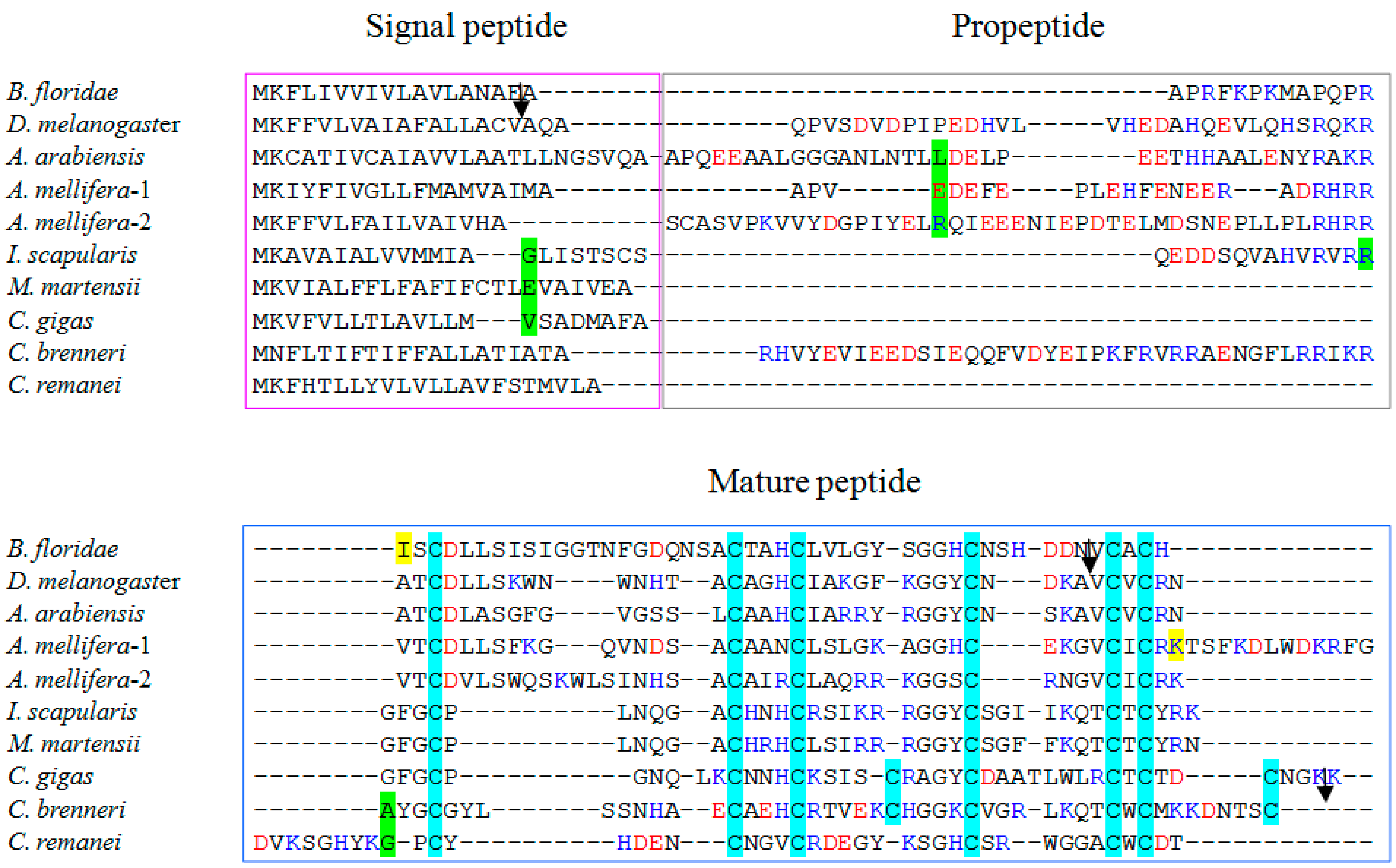

Supplement: Supplementary File 1 [file pharmaceuticals-07-00866-s001.pdf]
